# Serologic and molecular evidence of *Brucella ovis* infection in ovine and caprine flocks in the State of Minas Gerais, Brazil

**DOI:** 10.1186/s13104-016-1998-2

**Published:** 2016-03-26

**Authors:** Luciana Fachini Costa, Moisés Sena Pessoa, Laís Bitencourt Guimarães, Anne Karoene Silva Faria, Rodrigo Pereira Morão, Juliana Pinto da Silva Mol, Luize Néli Nunes Garcia, Anna Christina Almeida, Aurora Maria Guimarães Gouveia, Marcos Xavier Silva, Tatiane Alves Paixão, Renato Lima Santos

**Affiliations:** Departamento de Patologia, Instituto de Ciências Biológicas da Universidade Federal de Minas Gerais, CEP 31.270-901 Belo Horizonte, MG Brazil; Departamento de Clínica e Cirurgia Veterinárias, Escola de Veterinária da Universidade Federal Minas Gerais, CEP 31.270-901 Belo Horizonte, MG Brazil; Instituto de Ciências Agrárias da Universidade Federal de Minas Gerais, CEP 39.404-006 Montes Claros, MG Brazil; Departamento de Medicina Veterinária Preventiva, Escola de Veterinária da Universidade Federal Minas Gerais, CEP 31.270-901 Belo Horizonte, MG Brazil

**Keywords:** *Brucellaovis*, Rams, Goats, Brucellosis

## Abstract

**Background:**

*Brucella ovis* infection is one of the leading causes of sub fertility and infertility in ovine, been characterized mainly by epididymitis, orchitis and testicular atrophy in rams. This study aimed to determine the frequency of *B. ovis* positivity in rams and goats flocks in the State of Minas Gerais, Brazil, by agarose gel immunodiffusion (AGID), ELISA, Rose Bengal, PCR and bacteriological isolation as diagnostic tools.

**Findings:**

Serum and urine samples were collected from properties with sheep or goat flocks, or from properties with mixed flock. Out of 50 sheep flocks, 6 % (3/50) were seropositive by AGID while 4 % (2/50) were positive by urine PCR for *B. ovis*. Out of five goat farms, 20 % (1/5) were seropositive for *B. ovis* by AGID. Mixed flock farms had 11.1 % (2/18) of positivity by AGID. By ELISA, 19.5 % (8/41) of sheep properties and 61.1 % (11/18) of the properties with mixed flocks were positive for *B. ovis*. No samples were positive in the test of Rose Bengal, ruling out exposure to smooth LPS *Brucella* species (particularly *Brucella melitensis*) and indicating that the positive in the ELISA was associated with *Brucella* spp. LPS rough (presumably *B. ovis*). No urine sample from sheep or goat was positive by bacteriological isolation.

**Conclusions:**

Our results demonstrate serologic or molecular evidence of *B. ovis* infection in several rams and billy goats from meso-regions of the State of Minas Gerais, Brazil. Also, this study report the indirect ELISA as an important tool for the diagnosis of *B. ovis* infection, as indirect ELISA in this study demonstrated to be the most sensitive diagnostic method adopted.

## Background

*Brucella ovis* infection is one of the major causes of sub fertility or infertility in rams. It is clinically characterized by epididymitis, testicular atrophy and infertility [1, 2]. Natural *B. ovis* infection occurs mostly in sheep, although there are reports of infection in deer [3]. Goats may also acquire *B. ovis* infection when experimentally inoculated [4]. Semen from infected rams is the most important source of infection. Diagnosis of *B. ovis* infections is usually based on clinical examination, serology and bacterial isolation from semen samples [5]. Although experimental studies demonstrated that *B. ovis* can be equally detected in urine and semen samples by PCR or nested PCR [6, 7].

The State of Minas Gerais, located in the Southeastern region of Brazil, has 588,383.6 km^2^ divided in 12 meso-regions. A previous study identified 190 goat farms, 120 sheep farms and 91 farms with both goats and sheep in Minas Gerais [8]. Although goats are susceptible to *B. ovis* experimental infection [4], it is not clear whether goats can sustain infection in their flocks under natural conditions. Therefore, the goal of this study was to determine the frequency of naturally infected ovine and caprine flocks, either raised separately or together, in Minas Gerais.

## Methods

This study was performed in strict accordance with the recommendations in the Guide for the Care and Use of Laboratory Animals: Eighth Edition (published by the US National Research Council). All blood and urine sampling was performed by expert veterinarians, and every effort was made to minimize suffering. Although, the protocol was not formally approved by an ethical committee, we are unable to apply for retrospective approval in Brazil due to current legislation. However, we acknowledge that ethical approval should have been sought prior to the initiation of the study. All animal owners provided consent for their livestock to be included in this study.

Serum and urine samples were collected as previously described [6]. Serologic methods included agarose gel immunodiffusion (AGID), ELISA, and Rose Bengal, whereas urine samples were processed for bacterial isolation and DNA extraction for polymerase chain reaction (PCR) as previously described [6]. A commercially available AGID kit (TECPAR, Curitiba, Brazil) was used according to the manufacturer´s instruction. Sensitivity of this AGID protocol has been estimated to be 70.1 under conditions of experimental infection [13]; Indirect ELISA was performed as previous described [9] using a recombinant *B. ovis* BP26 antigen and serum dilutions of 1:20 and 1:40. Rose Bengal was performed following manufacture´s instruction (Instituto Biológico, São Paulo, Brazil) to detect antibodies against smooth *Brucella* LPS [10]. For bacterial isolation, 100 μL of urine samples were aseptically plated on modified Thayer Martin agar. Plates were incubated at 37 °C with 5 % CO_2_ for 7 days. PCR was performed by extracting DNA from 1 mL of urine. PCR reaction and cycling parameters were previously described [6]. Frequency of positivity by AGID, PCR, and ELISA were compared among farm groups (i.e. sheep, goat or mixed farms), and frequency of positivity of individual rams and billy goats were compared by Fisher’s exact test using GraphPad Instat software version 3.10. Differences were considered significant when P < 0.05.

The number of farms included in this study and their categories are detailed in Table 1. According to the “Instituto Brasileiro de Geografia e Estatística” [11], the ovine and caprine populations in the State of Minas Gerais are estimated to be 228,306 and 118,572, respectively, which corresponds to 1.3 % of the Brazilian population for both species. Blood and urine samples were collected from 124 rams and 34 billy goats, which considering the usual proportion of breeders in ovine and caprine flocks, supports the notion that between 1 and 2 % of the population of ovine and caprine breeders in the State of Minas Gerais have been sampled in this study. The experimental design was based on: (i) frequency of seropositivity for *B. ovis* of 5.3 % in the State of Minas Gerais [12]; (ii) sensitivity of 82 % and specificity of 98.8 %, in urine samples for the PCR assay used in this study [6]; (iii) a confidence interval of 95 % and statistic error of 5 %. Minas Gerais has a large territory and heterogeneous distribution of flocks, the number of farms to be sampled in each meso-region followed the records of the “Associação de Criadores de Caprinos e Ovinos de Minas Gerais” (Caprileite/ACCOMIG). “Campo das Vertentes” and “Noroeste” regions were not sampled due to the very small number of registered farms. One male breeder was sampled for each 25 females, up to eight male breeders per farm, and selection of animals for sampling was completely random within a flock. Sample sizes were based on parameters described above, and they were calculated using tools available at http://epitools.ausvet.com.au; which indicated a minimum number of 73 and 19 for ovine and caprine, respectively (124 rams and 34 billy goats were included in this study). The minimum numbers of flocks were estimated to be 32, 5, and 15 for ovine, caprine and mixed flocks, respectively (50 ovine, 5 caprine, and 18 mixed flocks were included in this study).

**Table 1:** Number of ovine, caprine, and mixed farms sampled in meso-regions of the State of Minas Gerais, Brazil

Meso-region	Ovine farms	Caprine farms	Mixed farms
Jequitinhonha	10	1	–
Norte de Minas	14	–	14
Vale do Mucuri	8	–	–
Central Mineira	4	–	–
Metropolitana de Belo Horizonte	2	3	–
Oeste de Minas	4	–	–
Sul/Sudeste de Minas	2	–	–
Triângulo/Alto Paranaíba	–	–	2
Vale do Rio Doce	5	–	2
Zona da Mata	1	1	–
Total	50	5	18

## Results

Farms that had at least one positive animal by any of the diagnostic method used were considered positive. Three of 50 ovine flocks sampled (6 %) were positive for *B.ovis* by AGID, while two (4 %) were positive by PCR of urine samples (Table 2). Positive farms were located in the “Vale do Jequitinhonha”, “Norte de Minas”, and “Oeste” meso-regions of Minas Gerais. One of five caprine farms sampled (20 %), located in the “Zona da Mata” meso-region, was positive for *B.ovis* by AGID. Two of 18 mixed flock (i.e. ovine and caprine) farms (11.1 %) were positive for *B.ovis* by AGID, one of which was located in “Norte de Minas” and the other in “Triângulo/Alto Paranaíba” meso-region (Table 2). In addition, some of the serum samples, that remained available after processing for AGID, were analyzed by a recently developed ELISA assay [9], and eight of 41 ovine farms (19.5 %) and 11 of 18 mixed flock farms (61.1 %) tested positive. ELISA positive ovine flocks were located in the “Vale do Jequitinhonha”, “Norte de Minas”, “Vale do Mucuri”, “Central Mineira”, and “Vale do Rio Doce” meso-regions, whereas ELISA positive mixed flocks were located in the “Norte de Minas” meso-region. None of the goat farms was positive for *B. ovis* by ELISA. There were no positive samples for *B. ovis* by bacterial culture of urine of rams (n = 124) and billy goats (n = 32) from ovine, caprine or mixed flock farms. Interestingly, considering all farms sampled, all PCR positive animals (ovine or caprine) were also serologically positive by ELISA, whereas none of the AGID positive animals (ovine and caprine) were positive in any of the other diagnostic methods employed in this study.

**Table 2:** Number of ovine, caprine, and mixed farms positive by agarose gel immunodiffusion (AGID), polymerase chain reaction (PCR) and ELISA for *Brucella* *ovis* in meso-regions of the State of Minas Gerais, Brazil

Meso-regions^a^	Positive ovine farms (%)^b^			Positive caprine farms (%)^b^			Positive mixed farms (%)^b^		
	AGID	PCR	ELISA	AGID	PCR	ELISA	AGID	PCR	ELISA
Jequitinhonha	20.0 (2/10)	0.0 (0/10)	11.1 (1/9)	0.0 (0/1)	0.0 (0/1)	0.0 (0/1)	–	–	–
Norte de Minas	0.0 (0/14)	14.2 (2/14)	28.5 (4/14)	–	–	–	7.1 (1/14)	0.0 (0/14)	78.5 (11/14)
Vale do Mucuri	0.0 (0/8)	0.0 (0/8)	12.5 (1/8)	–	–	–	–	–	–
Central Mineira	0.0 (0/4)	0.0 (0/4)	33.3 (1/3)	–	–	–	–	–	–
Metropolitana de Belo Horizonte	0.0 (0/2)	0.0 (0/2)	0.0 (0/1)	0.0 (0/3)	0.0 (0/3)	0.0 (0/3)	–	–	–
Oeste de Minas	25.0 (1/4)	0.0 (0/4)	–	–	–	–	–	–	–
Sul/Sudeste de Minas	0.0 (0/2)	0.0 (0/2)	0.0 (0/2)	–	–	–	–	**–**	**–**
Triângulo/Alto Paranaíba	–	–	–	–	–	–	50.0 (1/2)	0.0 (0/2)	0.0 (0/2)
Vale do Rio Doce	0.0 (0/5)	0.0 (0/5)	33.3 (1/3)	–	–	–	0.0 (0/2)	0.0 (0/2)	0.0 (0/2)
Zona da Mata	0.0 (0/1)	0.0 (0/1)	0.0 (0/1)	100.0 (1/1)	0.0 (0/1)	0.0 (0/1)	–	**–**	**–**
Total	6.0 (3/50)^a,b^	4.0 (2/50) ^a^	19.5 (8/41)^b*^	20.0 (1/5)^a^	0.0 (0/5)^a^	0.0 (0/5)^a^	11.1 (2/18)^a^	0.0 (0/18)^a^	61.1 (11/18)^b**^

Different letters in same category of flock indicate significant differences by Fisher exact test

* P < 0.05, **P < 0.01, ***P < 0.001

^a^“Campo das Vertentes” and “Noroeste” meso-regions were not sampled

^b^Data corresponds to percentage, with absolute positive/negative numbers indicated between parenthesis

Considering the total number of animals analyzed, three of 124 rams (2.4 %) were seropositive by AGID, while four (3.22 %) were positive by PCR of urine samples (Table 3). Three of 34 billy goats (8.8 %) were positive for *B. ovis* by AGID. Considering the ELISA results, 29 of 94 rams (30.8 %), and 11 of 31 goats (35.4 %) tested positive. Interestingly, 11 out of 17 billy goats (64.7 %) were positive for *B. ovis* by ELISA in the “Norte de Minas” meso-region. There was no statistical difference in the frequencies of positive ovine or caprine samples assessed by AGID, bacterial isolation or PCR (P > 0.05). The frequency of positivity was significantly higher by ELISA when compared to AGID or PCR in samples from rams and billy goats from several meso-regions in the State of Minas Gerais. None of the 94 ovine and 31 caprine serum samples was positive by Rose Bengal test.

**Table 3:** Number of rams and billy goats positive by agarose gel immunodiffusion (AGID), polymerase chain reaction (PCR), and ELISA for *Brucella* *ovis* in meso-regions of the State of Minas Gerais, Brazil

Meso-regions^a^	Positive rams (%)^b^			Positive billy goats (%)^b^		
	AGID	PCR	ELISA	AGID	PCR	ELISA
Jequitinhonha	15.3 (2/13)	0.0 (0/13)	9.0 (1/11)	0.0 (0/1)	0.0 (0/1)	0.0 (0/1)
Norte de Minas	0.0 (0/66)	6.0 (4/66)	45.4 (25/55)	5.0 (1/20)	0.0 (0/20)	64.7 (11/17)
Vale do Mucuri	0.0 (0/12)	0.0 (0/12)	11.1 (1/9)	–	–	–
Central Mineira	0.0 (0/4)	0.0 (0/4)	33.3 (1/3)	–	–	–
Metropolitana de Belo Horizonte	0.0 (0/4)	0.0 (0/4)	0.0 (0/2)	0.0 (0/5)	0.0 (0/5)	0.0 (0/5)
Oeste de Minas	12.5 (1/8)	0.0 (0/8)	–	–	–	–
Sul/Sudeste de Minas	0.0 (0/3)	0.0 (0/3)	0.0 (0/3)	–	–	–
Triângulo/Alto Paranaíba	0.0 (0/4)	0.0 (0/4)	0.0 (0/4)	33.3 (1/3)	0.0 (0/3)	0.0 (0/3)
Vale do Rio Doce	0.0 (0/9)	0.0 (0/9)	16.6 (1/6)	0.0 (0/3)	0.0 (0/1)	0.0 (0/3)
Zona da Mata	0.0 (0/1)	0.0 (0/1)	0.0 (0/1)	50.0 (1/2)	0.0 (0/2)	0.0 (0/2)
Total	2.4 (3/124)^a^	3.2 (4/124)^a^	30.8 (29/94)^b***^	8.8 (3/34)^a^	0.0 (0/32)^a^	35.4 (11/31)^b**^

Different letters in same animal species indicate significant differences by Fisher exact test

* P < 0.05, **P < 0.01, ***P < 0.001

^a^“Campo das Vertentes” and “Noroeste” meso-regions were not sampled

^b^Data corresponds to percentage, with absolute positive/negative numbers indicated between parenthesis

## Discussion

Although none of the samples yielded isolation of *B. ovis*, serologic analyses detected antibodies anti-*B. ovis* by AGID and indirect ELISA. In addition, *B. ovis* shedding in urine of rams was confirmed by PCR. These results are in good agreement with a previous study that demonstrated higher sensitivity of PCR and serology when compared to bacteriologic isolation even in experimentally infected rams [6, 13]. França et al. [9] reported sensitivity of 100 %, specificity of 90.2 % and accuracy equal to 1.0 for *B. ovis* detection by indirect ELISA using BP26r, which has been employed in this study, and therefore the possibility of a few false negative results should be considered while interpreting the results in the present study. Ours results demonstrated that the indirect ELISA had higher sensitivity when compared to AGID, which is know to have lower sensitivity [13]. None of the serum samples was positive by Rose Bengal test, indicating that the positivity obtained by the BP26 ELISA is indeed due to infection with rough *Brucella* sp. (i.e. *B. ovis*). Previous studies have demonstrated that seropositivity for *B. ovis* by AGID is intermittent throughout the course of infection. Indeed experimentally infected rams that shed *B. ovis* in the semen may have no antibody detectable by AGID [13, 14]. Interestingly, all PCR-positive animals in this study were also serologically positive by ELISA, while none of the AGID-positive animals were positive by any other diagnostic method. Poor agreement between AGID and PCR has been previously demonstrated under field conditions [15]. Furthermore, AGID has low sensitivity since experimentally infected rams that shed viable *B. ovis* in the urine and semen may be negative by AGID [6, 13].

The indirect ELISA had also higher frequency of seropositivity when compared to PCR of urine samples from rams. The PCR assay used in this study was previously developed by Xavier et al. [6]. This PCR protocol has a higher sensitivity than bacteriologic isolation from semen of naturally infected rams. Shedding of *B. ovis* in semen and urine of experimentally infected rams occurs intermittently [6], which explains the negative results obtained by PCR of urine samples from rams that were positive by indirect ELISA. Although semen samples have been traditionally employed for laboratorial diagnosis of *B. ovis* infection in rams, a previous study has demonstrated that efficacy of PCR detection of *B. ovis* genomic sequences is similar in semen and urine samples [6]. Therefore, urine samples were elected as the sample of choice since semen sampling under field conditions from rams and billy goats that were not properly conditioned would be technically challenging without expectations of better diagnostic results.

Higher frequencies of *B. ovis* positivity by ELISA and PCR were observed in the “Norte de Minas” meso-region of Minas Gerais. In addition, positive samples by ELISA from rams and billy goats from mixed farms were identified in the “Norte de Minas” meso-region. Domestic sheep is the preferential host for *B. ovis* infection, but *B. ovis* infection has been successfully induced by experimental inoculation of goats [4], white-tailed deer (*Odocoileus virginianus*), red deer (*Cervus elaphus elaphus*), and bighorn sheep [16–18]. Additionally, *B. ovis* transmission has been reported between sheep and red deer when sheeps infected are kept in the same paddock as non-infected red deers [3]. In spite of a comprehensive epidemiological study on seroprevalence of *B. ovis* infection in rams in the State of Rio Grande do Sul (Brazil) [19], to our knowledge these data reports the first evidence of antibody titers against *B. ovis* in naturally exposed goats. Higher frequency of *B. ovis* seropositivity was observed in billy goats kept in direct contact with sheep, in mixed flocks, which indicates that sheep is an important source for exposure of goats to *B. ovis*, although we found serologic evidences of exposure in goats raised in the absence of sheep.

## Conclusions

In conclusion, we identified rams and billy goats from several meso-regions of the State of Minas Gerais that had serologic or molecular evidences of *B. ovis* infection. Furthermore, the indirect ELISA was the most sensitive diagnostic method in the context of this study. Although these evidences of natural exposure and infection of goats with *B. ovis* were established in a restricted geographical area (i.e. the State of Minas Gerais, Brazil), these results have implications to all sheep and goat raising areas worldwide since diagnosis and control of *B. ovis* infection should ignore caprine as an alternative host. The absence of positive serologic results detecting smooth LPS has relevant significance since in contrast to *Brucella melitensis*, which has a smooth LPS and high zoonotic potential, the stably rough *B. ovis* is not associated with human infections.

## Abbreviations

ACCOMIG: “Associação de Criadores de Caprinos e Ovinos de Minas Gerais”
AGID: agarose gel immunodiffusion
*B. ovis*: *Brucella ovis*
CAPES: “Coordenação de Aperfeiçoamento de Pessoal de Nível Superior”
CNPq: “Conselho Nacional de Desenvolvimento Científico e Tecnológico”
FAPEMIG: “Fundação de Amparo à Pesquisa do Estado de Minas Gerais”
IBGE: “Instituto Brasileiro de Geografia e Estatística”
PCR: polymerase chain reaction
